# A Delphi Consensus of the Crucial Steps in Gastric Bypass and Sleeve Gastrectomy Procedures in the Netherlands

**DOI:** 10.1007/s11695-018-3219-7

**Published:** 2018-04-09

**Authors:** Mirjam A. Kaijser, Gabrielle H. van Ramshorst, Marloes Emous, Nic J. G. M. Veeger, Bart A. van Wagensveld, Jean-Pierre E. N. Pierie

**Affiliations:** 1 University of Groningen, University Medical Centre Groningen, Post Graduate School of Medicine, Groningen, The Netherlands; 2 Medical Centre Leeuwarden, Department of Surgery, Leeuwarden, The Netherlands; 3 Department of Surgery, The Netherlands Cancer Institute, Amsterdam, The Netherlands; 4 Department of Surgery, VU University Medical Center, Amsterdam, The Netherlands; 5 Department of Epidemiology, Medical Centre Leeuwarden, Leeuwarden, The Netherlands; 6 Department of Epidemiology, University of Groningen, University Medical Centre Groningen, Groningen, The Netherlands; 7 QURO Obesity Centers – Middle East, Dubai, United Arab Emirates; 8 Department of Surgery, OLVG West, Amsterdam, The Netherlands

**Keywords:** Delphi consensus, Procedural steps, Key steps, Bariatric surgery, Gastric bypass, Gastric sleeve

## Abstract

**Purpose:**

Bariatric procedures are technically complex and skill demanding. In order to standardize the procedures for research and training, a Delphi analysis was performed to reach consensus on the practice of the laparoscopic gastric bypass and sleeve gastrectomy in the Netherlands.

**Methods:**

After a pre-round identifying all possible steps from literature and expert opinion within our study group, questionnaires were send to 68 registered Dutch bariatric surgeons, with 73 steps for bypass surgery and 51 steps for sleeve gastrectomy. Statistical analysis was performed to identify steps with and without consensus. This process was repeated to reach consensus of all necessary steps.

**Results:**

Thirty-eight participants (56%) responded in the first round and 32 participants (47%) in the second round. After the first Delphi round, 19 steps for gastric bypass (26%) and 14 for sleeve gastrectomy (27%) gained full consensus. After the second round, an additional amount of 10 and 12 sub-steps was confirmed as key steps, respectively.

Thirteen steps in the gastric bypass and seven in the gastric sleeve were deemed advisable. Our expert panel showed a high level of consensus expressed in a Cronbach’s alpha of 0.82 for the gastric bypass and 0.87 for the sleeve gastrectomy.

**Conclusions:**

The Delphi consensus defined 29 steps for gastric bypass and 26 for sleeve gastrectomy as being crucial for correct performance of these procedures to the standards of our expert panel. These results offer a clear framework for the technical execution of these procedures.

## Introduction

Bariatric surgery is the golden standard for the treatment of morbid obesity because of its superior long-term results [1]. As these procedures aim not only to induce weight loss, but also to reduce comorbidity and increase life expectancy, high-quality standards are demanded by medical society, the public, and health authorities [2]. Multiple countries have adapted nationwide registries to ensure adequate auditing of surgical outcomes. Examples of these are the National Bariatric Surgery Registry (NBSR) in the UK, the Scandinavian Obesity Surgery Registry (SOReg), and the Dutch Audit for Treatment of Obesity (DATO) databases. These databases also provide opportunities to enhance these outcomes. Other improvement initiatives include peer review of technical skill and telementoring [3, 4].

However, a wide variation of techniques exists in literature, ranging from fully stapled to hand-sewn anastomosis techniques. This complicates comparing outcomes of scientific studies in terms of operating times, adverse events, and weight loss effects. Improvement of surgical quality may be achieved by offering detailed guidelines for the technical execution of surgical procedures. Standardization can also enhance training opportunities, facilitate feedback, and reduce error, resulting in shortening of the learning curve of these advanced laparoscopic procedures.

Khamis et al. defined the deconstruction of procedures into key steps as a part of the educational strategy and curriculum development [5]. The Delphi method is a well-described technique for obtaining consensus between groups of experts, which can easily be used by email questionnaires [6, 7]. Previous research has used the Delphi method to reach consensus on the key steps for appendicectomy, cholecystectomy, sigmoid resection, and right-sided colectomy [8, 9]. Coa et al. demonstrated hierarchical task analysis of surgical procedures such as cholecystectomy, inguinal hernia repair, and fundoplication. These procedures can be broken into surgical steps and sub-steps and tasks and sub-tasks, and these could even be divided into level of motions [10, 11].

The presented study aimed to reach expert consensus on the performance of the laparoscopic Roux-en-Y gastric bypass (LRGYB) and laparoscopic sleeve gastrectomy. These are the predominant bariatric procedures in the Netherlands, accounting for 89% of all primary procedures [12]. This consensus will be used in the development of a training and feedback program for bariatric surgery. For the purpose of creating a technical framework, this study identified the surgical steps and sub-steps as described by Coa et al. [10].

## Methods

### Participant Selection

Bariatric clinics’ websites were searched for names and contact details of all bariatric surgeons in the Netherlands. Moreover, an invitational email was send through the Dutch Society of Metabolic and Bariatric Surgery to lead surgeons from all bariatric centers and forwarded to their fellow bariatric surgeons. All 68 identified bariatric surgeons in the Netherlands were invited to participate in this consensus analysis.

### Step Identification

Through a literature search and operative protocols, the LRYGB and LSG procedures were divided into surgical steps. Next, these steps were broken down into a broad range of sub-steps. This study refrained from the task level, which would include, for example, introduction and extraction of separate instruments. As in previous research, the linear-stapled technique with suture closing of the remnant defect was most commonly used in the Netherlands [13].

### Delphi Processes

All bariatric surgeons received emails linked to a web-based questionnaire on SurveyMonkey^®^, asking to comment on the full list of steps in both LRYGB and LSG. In this first round, participants were asked to rate the different sub-steps on a 5-point Likert scale (not important, sometimes important, important, very important, essential). They were instructed to comment on a step if needed, regarding order, content, or even missing steps. Reminders were sent after 7 and 10 days. The 2-week response period was extended to 3 weeks to ensure the preset 50% participant response rate in the first round. After statistical analysis, sub-steps with a 95% confidence interval (CI) entirely < 3 were excluded as not relevant. Sub-steps with a direct CI > 4 were marked as key steps. All others were reevaluated in a second round, again with a 3-week response time. The same 5-point Likert scale was used, but respondents were allowed to comment on all sub-step responses and urged to comment on scores 1 and 2 (i.e., “not important” or “sometimes important”). Sub-steps with a complete 95% CI > 3.5 were again marked as key steps. Items with a mean > 3.5 were marked “advisable.” These criteria are summarized in Table 1. In line with earlier Delphi key step identification, it was hypothesized that two rounds would be sufficient for consensus [8, 9].

**Table 1:** Selection process based on the limits of the 95% confidence interval and means

	First round	Second round
Key step	Lower limit CI > 4	Lower limit CI > 3.5
Advisable	n.a	CI < 3.5; mean > 3.5
Reevaluation in second round	Lower and upper limit CI 3–4	n.a
Excluded/non-relevant	Upper limit CI < 3	CI < 3.5; mean < 3.5

*CI* confidence interval, *n.a* not applicable

### Statistical Analysis

Analysis was performed by SAS statistical software version 9.2. Consensus, or internal consistency, between experts was defined as a Cronbach’s alpha of at least 80% for each procedure. The responses of each sub-step were evaluated as continuous outcomes. Next, the correlations between the answers of the individual respondents were calculated for both procedures, as well as the overall correlations between all respondents, the Cronbach coefficient alpha. This analysis was repeated after the second round.

## Results

The survey of lead surgeons and website search resulted in the collection of contact details of 68 surgeons performing bariatric procedures, in 20 Dutch bariatric centers. A total of 38 surgeons participated (response rate 56%), representing 18 of the 20 clinics (90%).

### Gastric Bypass

The LRYGB procedure was divided into nine surgical steps: operative setup, starting laparoscopy, creating the pouch, creating the biliopancreatic limb, performing gastro-jejunostomy, creating the alimentary limb, performing entero-enterostomy, check of the bypass, and finishing the procedure. Next, the surgical steps were divided into 73 sub-steps. A complete list of these is represented in the first column of Table 2 [14–18].

**Table 2:**
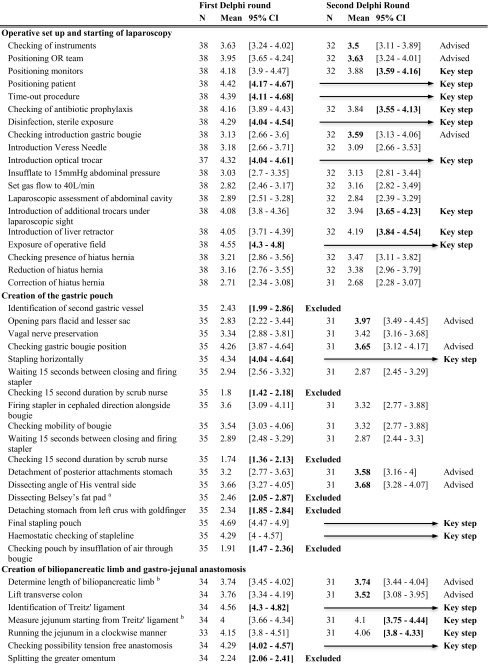

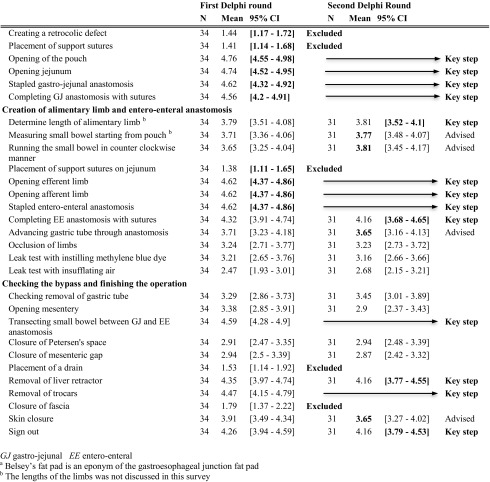
Delphi consensus on laparoscopic Roux-en-Y gastric bypass

Four out of 38 participants ended the survey prematurely. The expert group reached a Cronbach’s alpha consensus of 0.96 in the first round. Nineteen sub-steps were included as key steps after this first round as the lower bound of the 95% CI was > 4, meaning at least 95% of respondents found these steps very important or essential. Twelve of 73 sub-steps were deemed unnecessary as the upper bound of the 95% CI was < 3, meaning most participants found this task not or only sometimes important. The other sub-steps were reassessed in a second round. The already conclusive ratings are highlighted in bold in Table 2.

In the second round, 33 participants responded, all in full (100%). For ten sub-steps, the CI in the second round had a lower limit > 3.5 and were included as key steps, resulting in a total of 29 key steps. Thirteen steps had a mean > 3.5, meaning most participants found the sub-step at least “important”; these steps were included as “advisable.” The Cronbach’s alpha was 0.82 in the second round.

### Gastric Sleeve

The LSG was broken down into six surgical steps: operative setup, starting laparoscopy, mobilization of the greater curvature, stapling the sleeve, check of the sleeve, and finishing the procedure. The first two steps were very similar to the preparation of laparoscopy in LRYGB. The identified surgical steps were divided into 51 sub-steps, found in the first column of Table 3 [14, 18–21].

**Table 3:**
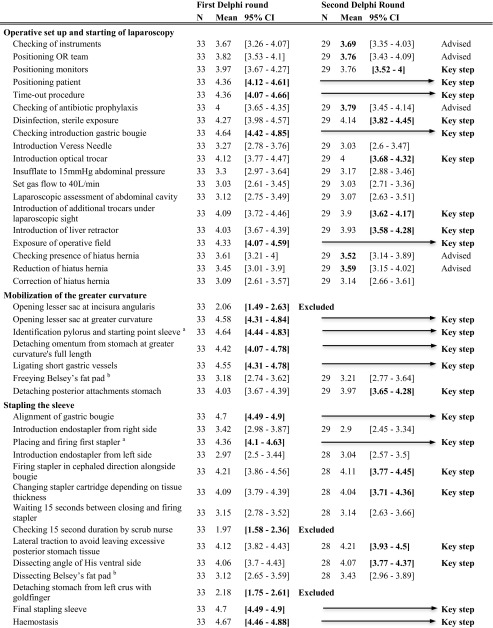

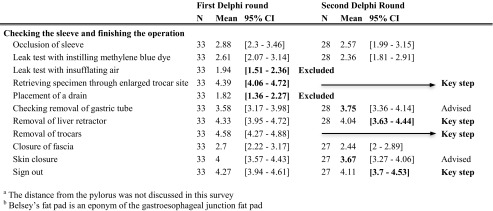
Delphi consensus on laparoscopic sleeve gastrectomy

Five participants indicated that LSG were not performed in their centers, leaving 33 participants (49%). In the first round, 14 steps (17%) obtained results with the entire 95% CI > 4; these were included as key steps. Five steps (10%) were excluded and 32 steps (63%) were reevaluated in a second round. A consensus with a Cronbach’s alpha of 0.95 was reached.

In the second round, 12 of the remaining items were accepted as key steps with a lower limit of the CI > 3.5, and the seven steps with a mean > 3.5 were deemed “advisable.” The other 13 sub-steps were excluded. The Cronbach’s alpha value was 0.87. The results of the Delphi analysis are displayed in Table 3.

For both procedures, this Delphi consensus resulted in a list of key steps and advised steps (Table 4). Due to the nature of the key step selection process, certain steps for both LRYGB and LSG required renaming. For example, the step “checking the bypass” contained six sub-steps. Only “transecting small bowel between gastro-jejunal and entero-enteral anastomosis” was marked as a key step, and this step was renamed “finishing the bypass” (see Table 4).

**Table 4:** Delphi consensus on laparoscopic gastric bypass and sleeve gastrectomy

Key step laparoscopic linear-stapled gastric bypass	Key step laparoscopic sleeve gastrectomy
Operative setup	Operative setup
*Checking of instruments*	*Checking of instruments*
*Positioning OR team*	*Positioning OR team*
Positioning monitors	Positioning monitors
Positioning patient	Positioning patient
Time-out procedure	Time-out procedure
Checking of antibiotic prophylaxis	*Checking of antibiotic prophylaxis*
Disinfection, sterile exposure	Disinfection, sterile exposure
*Checking introduction gastric bougie*	Checking introduction gastric bougie
Starting of laparoscopy	Starting of laparoscopy
Introduction optical trocar	Introduction optical troca
Introduction of additional trocars under laparoscopic sight	Introduction of additional trocars under laparoscopic sight
Introduction of liver retractor	Introduction of liver retractor
Exposure of operative field	Exposure of operative field
Creation of the gastric pouch	*Checking presence of hiatus hernia*
*Opening pars flacid and lesser sac*	*Reduction of hiatus hernia*
*Checking gastric bougie position*	Mobilization of the greater curvature
Stapling horizontally	Opening lesser sac at greater curvature
*Detachment of posterior attachments stomach*	Identification pylorus and starting point sleeve
*Dissecting angle of His ventral side*	Detaching omentum from stomach at greater curvature’s full length
Final stapling pouch	Ligating short gastric vessels
Hemostatic checking of staple line	Detaching posterior attachments stomach
Creation of biliopancreatic limb	Stapling the sleeve
Determine length of biliopancreatic limb	Alignment of gastric bougie
Lift transverse colon	Placing and firing first stapler
Identification of Treitz’ ligament	Firing stapler in cephaled direction alongside bougie
Measure jejunum starting from Treitz’ ligament	Changing stapler cartridge depending on tissue thickness
Running the jejunum in a clockwise manner	Lateral traction to avoid leaving excessive posterior stomach tissue
Gastro-jejunal anastomosis	Dissecting angle of His ventral side
Checking possibility for a tension free anastomosis	Final stapling sleeve
Opening of the pouch	Hemostasis
Opening jejunum	Finishing the sleeve
Stapled gastro-jejunal anastomosis	Retrieving specimen through enlarged trocar site
Completing gastro-jejunal anastomosis with sutures	Finishing the operation
Creation of alimentary limb	*Checking removal of gastric tube*
Determine length of alimentary limb	Removal of liver retractor
*Measuring small bowel starting from pouch*	Removal of trocars
*Running the small bowel in counter clockwise manner*	*Skin closure*
Entero-enteral anastomosis	Sign out
Opening efferent limb	
Opening afferent limb	
Stapled entero-enteral anastomosis	
Completing entero-enteral anastomosis with sutures	
*Advancing the gastric tube through anastomosis*	
Finishing the bypass	
Transecting small bowel between gastro-jejunal and entero-enteral anastomosis	
Finishing the operation	
Removal of liver retractor	
Removal of trocars	
*Skin closure*	
Sign out	

Advised steps are printed in italic

## Discussion

This study is as far as we know the first attempt to obtain a nationwide consensus of the performance of the gastric bypass and sleeve gastrectomy. In this discussion, we will critically review the used Delphi technique, evaluate the validity of the results by comparing those of other authors, and highlight parts of this consensus.

The use of the Delphi technique is widely recognized as a tool to obtain consensus between groups of experts, but the definition and composition of such an expert panel may affect the results. There are no exact rules described in literature for the composition of such an expert group [6, 7]. For this study, all surgeons who performed bariatric operations routinely and, thus, are stakeholders of the results of this consensus were invited to participate in the expert group. In this way, both surgeons who pioneered and surgeons with recent training could participate. The Delphi method itself ensures that all opinions can influence the consensus.

As surgeons from 90% of the Dutch bariatric centers participated in this study, the expert group can be considered to represent the Netherlands, and our preset goal of reaching a minimum 50% response rate in the first round was reached. The number of participants for the consensus in sleeve gastrectomy was lower, as this procedure is not performed in all centers. The Delphi methodology has the advantage of being performed by email, as the participants were selected from all of the Netherlands. A panel meeting was omitted for the reason of travel distance. To ensure the possibility of redefining the sub-steps after the first round, the participants were encouraged to comment on their rankings through the SurveyMonkey^®^.

A drawback of the used Delphi methodology is the “fatigue” of the respondents and declining of response rates, described to occur after two or three rounds. To minimize this effect, it was stated beforehand to use the expert panel two times. Zevin et al. also used the Delphi technique to gain expert consensus on the sub-steps of LRYGB [22]. With this consensus, the Bariatric Objective Structured Assessment of Technical Skills (BOSATS) was created. In the research of Zevin et al., two rounds were also sufficient for consensus. To optimize the results, a “pre-round” of selecting the possible steps from an extensive literature search was added, which is considered an acceptable strategy [7]. Nonetheless, the large number of sub-steps of the combined procedures may have influenced the results. For LRYGB, the sub-step “completing the pouch in a cephaled direction” was excluded, although the procedure cannot be done without this step. This suggested that either the inclusion criteria should be expanded, or participants may have found the sub-step too obvious.

Zevin et al. also performed a hierarchical task analysis to define the key steps of LRYGB [22]. Their analysis started with a total of 214 discrete steps and their results returned 99 sub-steps for review, with optional steps depending on the type of anastomosis. This difference can be explained by continuation of the hierarchical task into task level. Also, air or methylene blue leak testing and closure of the mesenteric defects were not considered common practice by members of the expert team and were omitted in the current analysis, resulting into fewer sub-steps. For the purpose of training and coaching in vivo, the sub-step level of the analysis may prove sufficient.

A recent study of Rutte et al. on the pitfalls of LSG identified only 13 key steps, half of the 26 key steps in this study [23]. This difference can be explained by the use of a hierarchical decomposition technique. As the six surgical steps in our study were broken into sub-steps, this may result into a more detailed list, not only regarding to the laparoscopic phase, but also including the start and end of the operation. Our expert panel excluded the 12th step described by Rutte et al., “closure of the left lateral port”. However, their first step “bupivacaine injection before trocar insertion” was not in our initial list of this study, but as more evidence has become available, this might be added as a key step [23–25].

For LRYGB, a high variety exists for the anastomosis techniques. Linear-stapled, circular-stapled, fully stapled, and hand-sewn techniques are described [14]. The tested list has the start of an antecolic omega loop bypass, with a linear-stapled technique for both the gastro-jejunal and entero-enteral anastomosis, resembling the simplified LRYGB as proposed by Ramos et al. [16]. Three respondents commented on this, reporting performance of a fully stapled technique or a circular-stapled method in which the sub-step “transecting small bowel between gastro-jejunal and entero-enteral anastomosis” occurred in an earlier stage of the procedure [26]. The Delphi technique was not used to provide consensus in the order of the performed steps, as these may be executed in a different sequence. These technical differences also explain why “completing gastro-jejunal anastomosis with sutures” in the LRYGB was accepted as a key step only in the second Delphi round, as some of the respondents used a stapler for this sub-step. Irrespective of the order and exact description of sub-steps, a high level of consensus was reached for both procedures, ranging from a Cronbach’s alpha between 0.82 and 0.96 in the first and second rounds. This demonstrates the reliability of the consensus.

The presented Delphi consensus showed that the expert panel considered the operative setup phase very important, as none of the proposed sub-steps were excluded in both LRYGB and LSG. In the second surgical step “starting laparoscopy,” sub-steps regarding handling of hiatal hernia were excluded for LRYGB, but advised for LSG. Some tested sub-steps such as “waiting 15 seconds between closing and firing stapler” and “checking 15 second duration by scrub nurse” depend on the use of specific instruments and should therefore have been regarded as tasks rather than sub-steps in hierarchical task analysis of these procedures.

The study was designed to not include most controversial sub-steps by, for example, not stating the lengths of the limbs for LRYGB. But the results of this study do highlight some of the current discussion topics in bariatric surgery such as the closure of mesenteric defects. This study shows that closing Petersen’s space and the defect between the entero-enteral anastomosis were not accepted as standard of care in the Netherlands at the time of the survey. However, some panelists remarked they were willing to change their standard procedure once more evidence on the benefits of closing the defects becomes available. For both procedures, leak tests with methylene blue or air were not considered a key step by this expert panel. It could be interesting to summarize this as “testing” in further research to ensure that some sort of testing is indeed not an advisable or key step.

While this study provides a consensus between Dutch surgeons of these specific operations, the results could serve as a basis for consensus in other countries and for different procedures such as the laparoscopic omega loop gastric bypass. The list of key steps can also be adjusted to incorporate different anastomosis techniques.

## Conclusion

Our Delphi analysis resulted in a list of 29 of 73 proposed steps of the LRYGB. Thirteen steps were deemed advisable. For the LSG, a list of 26 key steps was composed, accompanied by seven advised steps. Now that a comprehensive framework for the execution of these procedures has been established, these lists could be used for evaluation of skill acquisition and to perform further research on training of these procedures.

The results of this study will be used for the development of a bariatric surgery-training model or curriculum and can also be implemented as part of a telementoring program, as a guideline for privilege granting and as the basis of a structured skill assessment.
